# Vaccination of HIV-infected pregnant women: implications for protection of their young infants

**DOI:** 10.1186/s40794-016-0044-7

**Published:** 2017-01-06

**Authors:** Ziyaad Dangor, Marta C. Nunes, Gaurav Kwatra, Sanjay G. Lala, Shabir A. Madhi

**Affiliations:** 1 Medical Research Council: Respiratory and Meningeal Pathogens Research Unit, University of the Witwatersrand, Johannesburg, South Africa; 2 Department of Science and Technology/National Research Foundation: Vaccine Preventable Diseases, University of the Witwatersrand, Johannesburg, South Africa; 3 Department of Paediatrics and Child Health, Faculty of Health Sciences, University of the Witwatersrand, Johannesburg, South Africa; 4 National Institute for Communicable Diseases: a division of National Health Laboratory Service, Johannesburg, South Africa

**Keywords:** Pregnant women, HIV, Antibody, Immunity, Vaccine

## Abstract

**Background:**

The prevention of mother to child transmission of HIV has resulted in reduced burden of pediatric HIV-infection, but the prevalence of maternal HIV infection remains high in sub-Saharan African countries. HIV-exposed-uninfected infants have an increased risk of morbidity and mortality due to infectious diseases than HIV-unexposed infants, particularly during the first six months of life, which in part might be due to lower levels of pathogen-specific protective antibodies acquired transplacentally from their mothers. This could be mitigated by vaccinating pregnant women to boost antibody levels; although vaccine responses among HIV-infected pregnant women might differ compared to HIV-uninfected women. We reviewed studies that compared natural and vaccine-induced antibody levels to different epitopes between HIV-infected and HIV-uninfected pregnant women.

**Findings:**

Most studies reported lower baseline/pre-vaccination antibody levels in HIV-infected pregnant women, which may not be reversed by antiretroviral therapy during pregnancy. There were only few studies on vaccination of HIV-infected pregnant women, mainly on influenza virus and group B *Streptococcus* (GBS) vaccines. Immunogenicity studies on influenza vaccines indicated that HIV-infected pregnant women had lower vaccine induced hemagglutination inhibition antibody titers and a decreased likelihood of seroconversion compared to HIV-uninfected women; and while higher CD4+ T-lymphocyte levels were associated with better immune responses to vaccination, HIV viral load was not associated with responses. Furthermore, infants born to influenza vaccinated HIV-infected pregnant women also had lower antibody levels and a lower proportion of HIV-exposed infants had titers above the putative correlate of protection compared to HIV-unexposed infants. The immunogenicity of a CRM_197_-conjugated trivalent GBS vaccine was also lower in HIV-infected pregnant women compared to HIV-uninfected women, irrespective of CD4+ T-lymphocyte counts.

**Conclusions:**

Poorer immunogenicity of vaccines reported in HIV-infected compared to HIV-uninfected pregnant women might compromise the potential benefits to their young infants. Alternate vaccination strategies, including vaccines with higher antigen concentration, adjuvanted vaccines or multiple doses schedules might be required in HIV-infected pregnant women to optimize antibody transferred to their fetuses.

## Background

### HIV-exposed infants, a new vulnerable population

Advances in the prevention of mother to child transmission (PMTCT) of HIV-1 infection have substantially decreased the prevalence of pediatric HIV-infection and the burden of HIV attributable-childhood infectious diseases, including in high prevalence sub-Saharan African countries [1]. Nevertheless, the prevalence of HIV among pregnant women remains high in many African countries, and there is emerging recognition of higher morbidity and mortality among HIV-exposed uninfected (HEU) than HIV-unexposed young infants [2–4]. Marinda et al. reported a 2.8-fold (95% CI: 2.1–3.9) and 5.5-fold (95% CI: 3.9–7.9) higher mortality rate among HEU compared to HIV-unexposed infants from birth to two months, and from two to six months of age, respectively [5]. Furthermore, HEU infants under six months of age have a 2.7-fold increased risk of invasive *Streptococcus pneumoniae* disease and 2.3-fold increased risk of invasive group B *Streptococcus* (GBS) disease [6, 7], which correlates to the degree of maternal immunosuppression [8]. Additionally, HEU compared to HIV-unexposed infants have 1.4-fold increased risk for hospitalization for common respiratory virus associated pneumonia, including respiratory syncytial virus and human metapneumovirus [9] (Table 1).

**Table 1:** Incidence ratio ratio’s and mortality in HIV-exposed and –unexposed infants less than 6 months of age

	Year	Incidence (95% CI) per 100 000		Incidence rate ratio (95% CI)	Mortality risk
		HIV-exposed	HIV-unexposed		
Invasive disease					
*Streptococcus pneumoniae* [6]	2013	57 (46–71)	21 (17–36)	2.7 (2.0–3.7)	1.8 (1.1–2.9)^a^
*Streptococcus agalactiae* ^b^ [7]	2004–2008	4.5 (3.9–5.1)	2.0 (1.7–2.3)	2.3 (1.9–2.8)	-
Lower respiratory tract associated hospitalization					
Respiratory syncytial virus [9]	2010–2011	5003 (4505–5541)	3507 (3244–3787)	1.4 (1.3–1.5)	2.1 (1.1–3.8)^c^
Human metapneumovirus [9]	2010–2011	816 (622–1050)	573 (470–692)	1.4 (1.1–2.0)	-

^a^adjusted relative risk ratio in HEU infants (37% [59/175]) versus HIV-unexposed infants (32% [51/228]) for 2009 and 2013; ^b^Less than 3 months of age; ^c^Odd ratio of death in HEU versus HIV-unexposed infants

The possible mechanisms for the increased susceptibility of HEU infants to infectious diseases were recently reviewed [3, 4, 10]. This included lower levels of pathogen-specific protective antibodies among HIV-infected women and reduced efficiency of transplacental transfer of antibodies. The lower epitope-specific antibody concentrations against vaccine preventable diseases in HIV-infected women [11–13], is likely due to HIV-induced loss of memory T- and B-lymphocyte function. Furthermore, placental abnormalities and saturation of active transport receptors may also result in inefficient transplacental antibody transfer to fetuses of HIV-infected women [10]. Compounding this might be immune dysregulation such as T-cell activation, increased pro-inflammatory cytokines secretion, and increased lymphocyte apoptosis in HEU infants [10]. Reduced thymic sizes and lower CD4+ T-lymphocyte counts were also observed in HEU infants. Although not clearly understood, *in-utero* antiretroviral therapy (ART) exposure has been associated with mitochondrial toxicity, lower numbers of circulating T-cell lymphocytes and neutrophils in young infants [10].

### Maternal vaccination as a strategy to prevent infant disease

Vaccination during pregnancy could potentially improve maternal and child health [14], as already shown by the effectiveness of vaccination during pregnancy with tetanus toxoid vaccine in reducing mortality from neonatal tetanus in low-middle income countries by >80% from proximately 1.27 million cases in the 1980s to <50,000 cases by 2013 [15]. Also, in the USA and some European countries, maternal vaccination strategies have been adopted for the prevention of influenza and pertussis in the women and their young infants [16]. There is growing public awareness about the benefits of maternal vaccination; with 72% of women in the United Kingdom considering vaccination during pregnancy as acceptable [17].

The protection of the infants could either be due to prevention of mother-to-child transmission of pathogens during close contact, or through transfer of maternal epitope-specific protective antibodies via the placenta and/or breastmilk. This is especially beneficial against diseases presenting soon after birth, or during the initial vulnerable period prior to young infants completing their immunization against vaccine preventable diseases [12]. The effectiveness of vaccination of pregnant women in protecting their infants through transplacental antibody transfer depends on a number of factors such as: (i) the immunogenicity of the vaccine among pregnant women, (ii) baseline maternal antibody levels and underlying prevalence of memory lymphocytes, (iii) subclass of the antibodies induced by the vaccine, (iv) efficiency of transplacental antibody transfer, (v) adequate gestational time to allow for optimal in-utero transplacental transfer of antibodies, and (vi) antibody half-life in the women and infant [18].

The transplacental acquired antibodies in the fetus are almost exclusively IgG antibodies, with more efficient and abundant transfer of IgG1 compared to IgG2 [18]. IgG2 is preferentially induced by polysaccharide epitopes, used in many vaccines against encapsulated organisms such as *Streptococcus pneumoniae*, GBS and *Haemophilus influenzae* type b; whereas IgG1 is induced primarily by protein epitopes [14, 18]. The transfer of maternal antibodies across the placenta is via an active transport mechanism utilizing Fc receptors [19]. To transfer from the maternal to the fetal circulation, IgG need to cross over the syncytiotrophoblast and the fetal endothelium [20]. Factors that might influence this transplacental antibody transfer include placental integrity, the IgG subclass, maternal nutritional status, parity/gravidity and gestational age [11, 14, 21].

Vaccination during pregnancy aimed at protecting the infants, needs to induce sufficient maternal antibody levels that will be transferred to the fetus within the remaining gestational period; and persist in the infant through early infancy. The active transfer of maternal IgG antibodies (natural or vaccine-induced) occurs most efficiently in the third trimester of pregnancy [22]. Infant to maternal antibody ratios are variable for different antigens, estimates generally range between 75 to 135% at term (≥37 weeks), 50–95% at 33–36 weeks, and 30–55% at 28–32 weeks gestation age [22–24]. Although a more efficient transfer of antibodies occurs in the last weeks before birth, infants born to women vaccinated in the second trimester had higher levels of pertussis antibodies than those vaccinated in the third trimester [25]. Generally, maternal and cord blood antibody concentrations are correlated; though higher maternal antibody concentrations and more efficient transplacental transfer are noted in mothers in high-income countries compared to low-income countries [11, 26]. This difference is most probably attributable to the higher prevalence of chronic maternal infections, placental infections or undernutrition in low-income countries [11, 27].

### Maternal HIV-infection and vaccination

**Fig 1:**
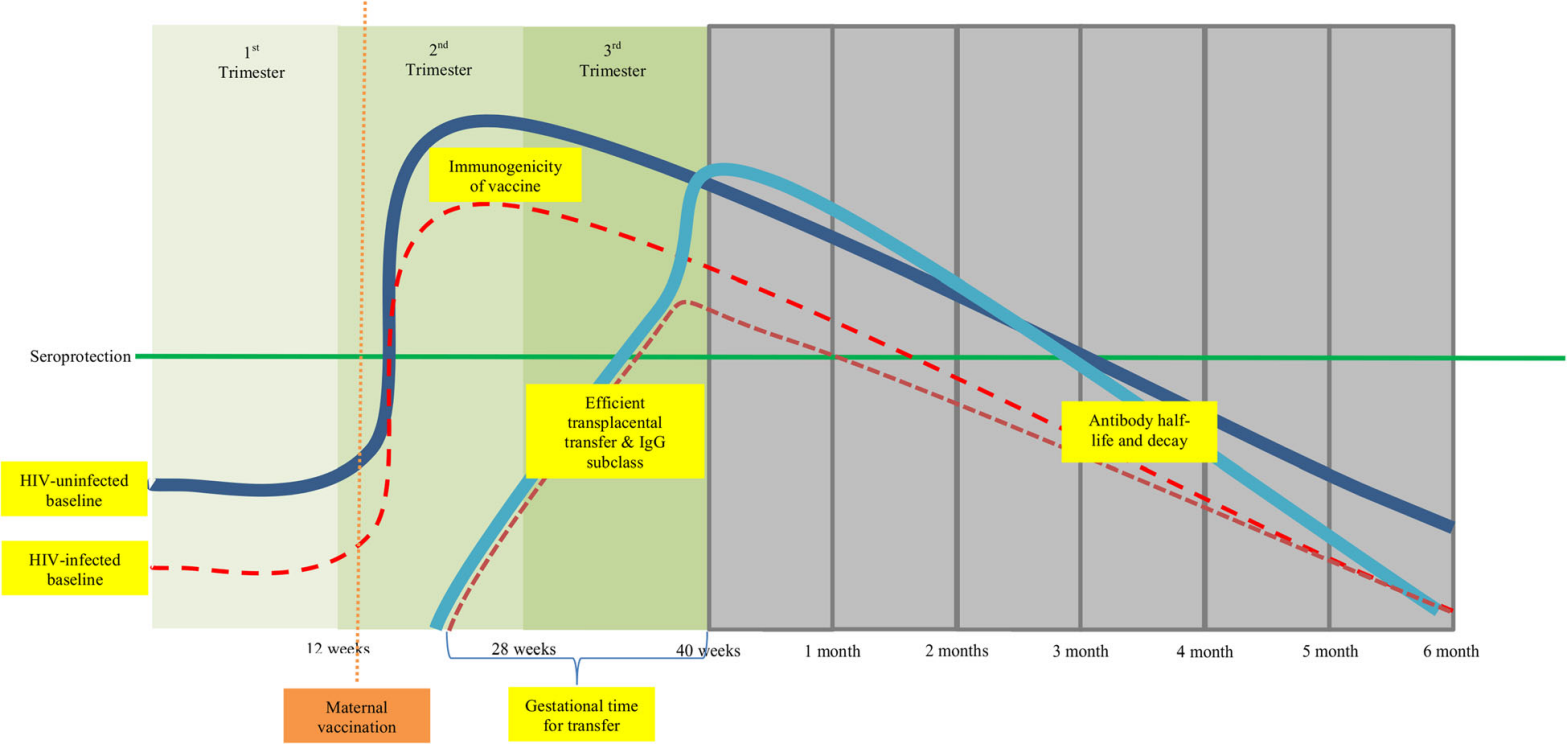
Proposed differences in vaccine-induced antibody responses between HIV-infected and –uninfected mother-infant pairs. Footnote: HIV-infected women [red dotted], HIV-uninfected women [dark blue], HIV-exposed infant [maroon dotted], HIV-unexposed infant [light blue]

Generally, HIV-infected pregnant women have lower baseline/pre-vaccination antibody levels, including to epitopes of measles virus, influenza virus, *Streptococcus pneumoniae*, *Haemophilus influenzae* type b, GBS and tetanus compared to HIV-uninfected women [11–13, 21, 28–30]. This is most likely due to a loss of epitope-specific T- and B-memory cells caused by the immunosuppressive progression of HIV-infection. Because most HIV-infected women of child-bearing age in low-middle income countries are only initiated on ART during pregnancy [1], the net loss of epitope-specific memory might already have occurred in those with more advanced disease and therefore late-ART initiation may not result in complete immune reconstitution [31]. An association between higher maternal CD4+ T-lymphocyte counts and maternal antibody baseline levels has been observed in women [12]. Therefore, unless the immune-status is preserved by early-ART, HIV-infected pregnant women will likely have lower pre-vaccination/baseline antibody levels and might mount poorer booster responses to vaccination [32] (Fig. 1).

This could be further compounded by reduced transplacental antibody transfer in HIV-infected compared to HIV–uninfected mother-newborn dyads (Fig. 1). HIV-infection is associated with a hyper-gammaglobulinemia state which might result in saturation of the Fc receptors, hence impeding antibody transfer to the fetus [20]. Studies have reported lower newborn to maternal antibody ratios for measles, varicella, tetanus, *Haemophilus influenzae*, pertussis and pneumococcus antibodies in HIV-infected compared to HIV-uninfected mother-newborn dyads [12, 13, 20, 21, 33]. Although a South African study did not identify an association between transplacental antibody transfer and CD4+ T-lymphocyte counts or HIV-1 viral load [12]; high maternal HIV-1 viral load was reported to reduce transplacental antibody transfer in another study [34].

#### Influenza vaccine and HIV-infected pregnant women

HIV-exposed uninfected infants less than six months have an increased risk of hospitalization and death from respiratory virus-associated lower respiratory tract infections, including influenza virus [2, 9]. In South Africa, two double-blind, randomized, placebo-controlled trials evaluated the safety, immunogenicity and efficacy of a trivalent influenza vaccine in HIV-infected and HIV-uninfected pregnant women, including follow-up of their infants until six months of age [35]. Vaccination of pregnant women with a trivalent influenza vaccine has proven to be safe for both the women and their infants [35]. Before vaccination and one month post-vaccination with the trivalent influenza vaccine, HIV-infected compared to HIV-uninfected pregnant women, had lower hemagglutination-inhibition (HAI) antibody titers and a decreased likelihood of seroconversion (41% vs. 92%, respectively to at least one strain) [30]. Similar observations were reported from a smaller study in the USA, where increased regulatory T-cells numbers were associated with attenuated immune responses to influenza vaccination in HIV-infected women, resulting in reduced immunogenicity to at least one influenza vaccine strain despite similar pre-vaccination HAI titers compared to HIV–uninfected women [36]. Interestingly, high pre-vaccine HAI titers correlated with lower seroconversion rates in HIV-uninfected women, whereas, high pre-vaccine titers correlated with improved seroconversion rates in HIV-infected women [30]. Correlation analyses of post-vaccination HAI titers and HIV viral load in HIV-infected pregnant women, most of whom were on ART, generally have not shown any association [30, 36–38]. Improved immune responses were, however, detected against at least one strain in the trivalent influenza vaccine in women with CD4+ T-lymphocyte counts of >350 cells/μL [30].

After vaccination with an unadjuvanted 2009 pandemic H1N1 monovalent influenza vaccine, HAI titers were also significantly increased in women with higher CD4+ T-lymphocyte counts [37, 38]. In the study of the 2009 pandemic, H1N1 monovalent influenza vaccination of 130 HIV-infected pregnant women, a second dose of a high-concentration (30 μg/dose) vaccine marginally improved the percentage of women with HAI titers above the putative seroprotective threshold of ≥1:40 and who demonstrated seroconversion (73% and 66% after dose 1 and 80% and 72% after dose 2, respectively). Nevertheless, HIV-infected women still had poorer immune responses than described in HIV-uninfected women [38]. Furthermore, non-pregnant HIV-infected adults had in general poorer immune responses to standard dose (15 μg) of unadjuvanted influenza vaccines compared to HIV-uninfected individuals [39–42].

The inferior immune response to unadjuvant inactivated influenza vaccines in HIV-infected adults has led to investigation for alternate strategies to improve the immune responses to vaccination in this population. Influenza vaccines with higher antigen content elicited higher HAI titers and improved seroresponse rates in HIV-infected non-pregnant adults, however, the responses were still inferior compared to HIV-uninfected adults [43, 44]. Furthermore, a second vaccine dose did not consistently increase the immune responses [40, 41, 43, 45]. Although the safety and immunogenicity of adjuvanted pandemic H1N1 vaccines has not been evaluated in HIV-infected pregnant women, immune responses in non-pregnant HIV-infected adults were better with adjuvanted pandemic H1N1 vaccine compared to unadjuvanted vaccines; this included higher percentage of ASO3-adjuvanted vaccine recipients developing HAI titers ≥1:40 compared to unadjuvanted vaccine–recipients [41]. HAI titers ≥1:40 were 70–93% and increased to 94–99% after 2 doses of the ASO3-adjuvanted vaccine, which was similar to the response described in HIV-uninfected individuals (>95%) [41, 46]. Also, a single dose of a MF59-adjuvanted vaccine was associated with 88% of HIV-infected adults developing HAI titers ≥1:40, which had a small increase to 91% following the second vaccination, which was similar to HIV-uninfected controls [47]. Overall, the HAI GMT titers were, however, lower in HIV-infected than HIV-uninfected adults [47].

**Fig. 2:**
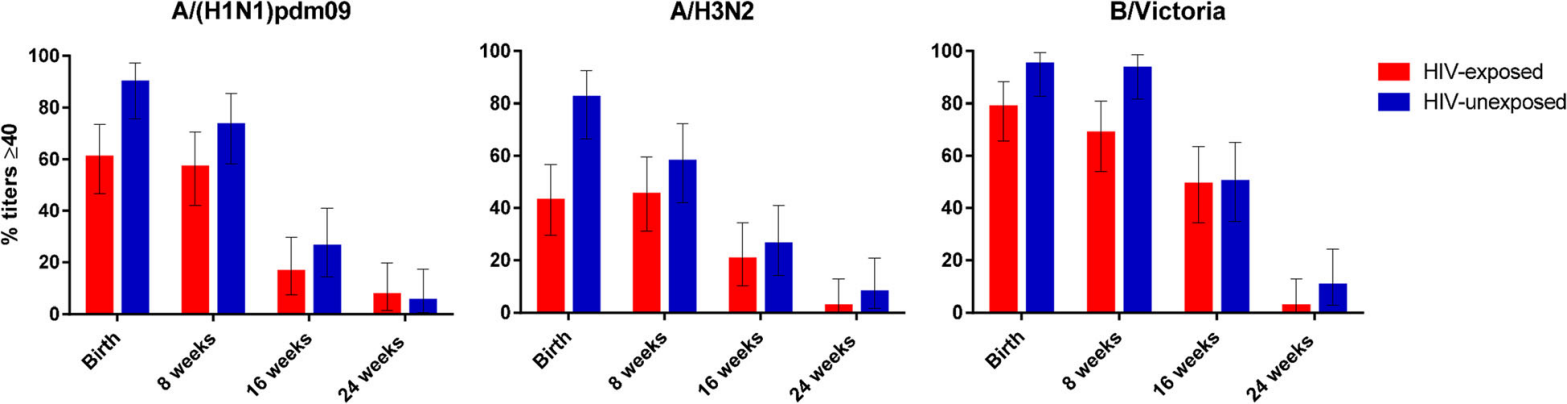
**a**, **b**, **c** Percentage of HIV-exposed and –unexposed infants with seroprotective antibody titers against the three influenza strains after their mothers had been vaccinated with influenza vaccine during pregnancy. Data from the maternal influenza South African clinical trial [50]

Among the newborns of HIV-infected women, birth HAI titers correlated strongly with maternal antibody titers at delivery [30, 38]. A comparison between HIV-infected and HIV-uninfected pregnant women vaccinated with trivalent influenza vaccine reported lower transplacental HAI antibody transfer in HIV-infected women for only one of the three vaccine strains [30]. Nevertheless, due to the lower HAI titers post-vaccination among HIV-infected pregnant women, their newborns had lower HAI antibody titers and were less likely to have HAI titers ≥1:40 compared to HIV-unexposed infants (ranging from 43–79% vs. 82–95%, respectively for the different vaccine strains) [30]. Despite the antibody half-life being longer in HIV-exposed (56–65 days) than in HIV-unexposed infants (43–45 days) [30], seroprotective levels did not persist in most infants beyond three months of age [38, 48] (Fig. 2). No direct comparison is available for infant HAI titers after maternal H1N1 monovalent influenza vaccination between HIV-exposed than in HIV-unexposed infants [38].

In healthy adults, a HAI titer of 1:40 or higher is associated with 50% protection against influenza disease [49]. However, data from studies on HIV-infected pregnant and non-pregnant adults suggest that influenza vaccination may confer protection to this population by additional mechanisms and that the use of HAI titers as a correlate of protection needs to be validated in these individuals [30, 50]. Furthermore it has been proposed that higher HAI titers may be required to provide protection against influenza in children [51].

In the South African randomized-controlled trials of trivalent influenza vaccine in pregnant women, the vaccine efficacy against PCR-confirmed influenza was similar in HIV-infected (58%; 95% CI: 0.2, 82) and HIV-uninfected (50%; 95% CI: 15, 71) women despite HIV-infected women having a reduced immune response to vaccination [30, 35]. Two other randomized-controlled trials evaluated the efficacy of trivalent influenza vaccine in pregnant women against influenza infection in the women and their infants until six months of age; however, the only study that measured vaccine efficacy in HIV-infected women was conducted in South Africa [35, 52, 53]. Incidentally, the trial conducted in Bangladesh reported fewer episodes of influenza infection during the first six months of life in infants born to influenza vaccinated pregnant women compared to infants born to women who received 23-valent pneumococcal vaccine; for laboratory confirmed influenza infection in infants the vaccine efficacy was 63% (95% CI: 5, 85) [52]. The two other larger African trials corroborated the protection afforded by maternal vaccination against PCR–confirmed influenza in infants younger than six months. In Mali infants born to women who received influenza vaccine had a lower influenza attack-rate compared to those born to women vaccinated with quadrivalent meningococcal vaccine, with an overall vaccine efficacy of 33% (95% CI: 4, 54) [53]. In South Africa the vaccine efficacy against PCR–confirmed influenza was 49% (95% CI: 12, 70) among infants born to HIV-uninfected women and while the study was not powered to detect vaccine efficacy in the HIV-exposed infants a modest vaccine efficacy point estimate was reported (26.7%; CI: −132, 77) [35]. Based on the immunogenicity data from the two South African trials, despite vaccine efficacy in the HIV-infected mothers (which was similar compared to HIV-uninfected women), alternate strategies to protect the HIV-exposed infants need to be considered. Whilst vaccine induced cell mediated immunity might play a role in adults, protection in the infants will only be mediated by the presence of antibodies; hence higher antibody concentrations need to be elicited in the women.

#### Group B Streptococcus and maternal HIV-infection

Although recto-vaginal GBS colonization is similar or lower in HIV-infected compared to HIV-uninfected pregnant women [54–56], HEU have a 2–20 fold increase susceptibility of invasive GBS disease compared to HIV-unexposed infants; and particularly so for late-onset disease (disease occurring from 7 to 89 days of life, LOD; 4–125 fold) [7, 57, 58]. Furthermore, HEU compared to HIV-unexposed infants with invasive GBS disease are more likely to present with meningitis than sepsis [58].

Natural serotype-specific and surface-protein IgG antibodies have been compared between HIV-infected and HIV-uninfected mother-newborn dyads [28, 29]. In two studies conducted in two different regions of South Africa with a high HIV prevalence, serotypes Ia, Ib, II, III and V-specific IgG antibody levels were consistently lower in HIV-exposed compared to HIV–unexposed newborns [28, 29]. This was likely due to the combination of lower maternal antibody concentrations and reduced transplacental antibody transfer between HIV-infected compared to HIV-uninfected mother-newborn pairs. Both studies, although not specifically powered to address the question, reported no associations between the maternal level of immune suppression and anti-GBS epitope antibody levels or transplacental ratios, although most women had CD4+ T-lymphocyte counts >350 cells/mL and were on ART during pregnancy [28, 29]. Furthermore, another recent study from South Africa showed that the proportion of individual IgG subclasses transferred through the placenta was unaffected by maternal HIV infection, with the exception of serotypes III IgG1; and importantly there was no difference in transplacental transfer of IgG2, the main subclass against GBS polysaccharides [59].

Maternal GBS vaccination is potentially cost effective, both in high income [60, 61] and low-middle income countries [62]. Early experimental conjugate vaccines induced good IgG responses in pregnant women which persisted for long periods and could also protect against LOD [63, 64]. Although there is no licensed GBS vaccine, an investigational trivalent GBS polysaccharide-protein conjugate vaccine that includes serotypes Ia, Ib and III was recently evaluated in a phase-II study among HIV-uninfected and HIV-infected pregnant women [65, 66]. In Malawi (18% maternal HIV prevalence) and South African (30% maternal HIV prevalence), lower vaccine immunogenicity was observed among the HIV-infected compared to HIV-uninfected pregnant women [66]. Among HIV-infected women, immune responses did not differ between those with low (50–350 cells/mL) and high (>350 cells/mL) CD4+ T-lymphocyte counts. Furthermore, antibody concentrations post-vaccination were lower in women without detectable antibody levels at baseline, suggesting an absence of memory lymphocytes in these women. Transplacental antibody transfer and antibody decay was similar between HIV-infected and HIV-uninfected mother-newborn dyads, nevertheless, HIV-exposed infants had lower antibody concentrations than HIV-unexposed infants at birth and at 6 weeks of age [66]. Because a correlate of protection has not been established, it is unknown whether antibody concentrations in HIV-exposed infants are sufficient to protect against invasive GBS disease.

## Conclusions

Antibody transfer from mother to fetus is crucial in protecting young infants against various infectious diseases prior to their primary immunization or during the period that they are unable to mount a robust immune response to infection. Poor immune responses to natural infection result in lower antibody levels in HIV-infected women and may contribute to disease susceptibility in HIV-exposed infants. Although maternal vaccination reduces early infant disease, lower immune responses to vaccines have been reported in HIV-infected compared to HIV-uninfected pregnant women; this needs to be considered in vaccine development, especially in settings with high maternal HIV-infection prevalence and regardless of PMTCT or ART. Females who were born with HIV and those on long-term ART would soon be entering child-bearing age; these women need to be further studied as to whether T- and B-cell memory is preserved.

Immunogenicity may be enhanced in HIV-infected women through two-dosing vaccine schedules [38]. The first dose would “re-prime” the HIV-infected women’s immune system and then the second dose would serve as a booster. Higher antigen concentrations and the use of conjugate vaccines have also shown improved immunogenicity and efficacy in HIV-infected adults and may be explored in HIV-infected pregnant women [38, 67]. The use of adjuvants to boost immune responses needs further investigation. Maternal vaccine immunogenicity studies or phase IV efficacy trials, coupled with cost-effectiveness studies must be conducted in HIV-infected pregnant women in highly endemic settings.

## Abbreviations

ART: Antiretroviral therapy
GBS: Group B Streptococcus
HEU: HIV-exposed uninfected
LOD: Late-onset disease
PMTCT: Prevention of mother to child transmission
